# Blood group AB increases risk for surgical necrotizing enterocolitis and focal intestinal perforation in preterm infants with very low birth weight

**DOI:** 10.1038/s41598-021-93195-8

**Published:** 2021-07-02

**Authors:** I. Martynov, W. Göpel, T. K. Rausch, C. Härtel, A. Franke, A. R. Franz, D. Viemann, U. H. Thome, M. Lacher, B. W. Ackermann

**Affiliations:** 1 Department of Pediatric Surgery, University of Leipzig, Liebigstraße 20 a, 04103 Leipzig, Germany; 2 Department of Pediatrics, University of Lübeck, Lübeck, Germany; 3 Institute for Medical Biometry and Statistics, University of Lübeck, Lübeck, Germany; 4 Institute of Clinical Molecular Biology, Christian-Albrechts-University of Kiel & University Hospital Schleswig-Holstein, Kiel, Germany; 5 Department of Neonatology, University Children’s Hospital Tübingen, Tübingen, Germany; 6 Center for Pediatric Clinical Studies (CPCS), University Children’s Hospital Tübingen, Tübingen, Germany; 7 Department of Pediatric Pneumology, Allergology and Neonatology, Hannover Medical School, Hannover, Germany; 8 Division of Neonatology, Center for Pediatric Research Leipzig, Hospital for Children and Adolescents, University of Leipzig, Leipzig, Germany

**Keywords:** Medical research, Paediatric research

## Abstract

Necrotizing enterocolitis (NEC) and focal intestinal perforation (FIP) are two of the most common emergencies of the gastrointestinal tract in preterm infants with very low birth weight (VLBW, birth weight < 1500 g). Identification of risk factors among these children is crucial for earlier diagnosis and prompt intervention. In this study, we investigated a relationship between ABO blood groups and the risk for surgical NEC/FIP. We genotyped the ABO locus (rs8176746 and rs8176719) in VLBW infants enrolled in a prospective, population-based cohort study of the German Neonatal Network (GNN). Of the 10,257 VLBW infants, 441 (4.3%) had surgical NEC/FIP. In univariate analyses, the blood group AB was more prevalent in VLBW infants with surgical NEC/FIP compared to non-AB blood groups (OR 1.51, 95% CI 1.07–2.13, p = 0.017; absolute risk difference 2.01%, 95% CI 0.06–3.96%). The association between blood group AB and surgical NEC/FIP was observed in a multivariable logistic regression model (OR of 1.58, 95% CI 1.10–2.26, p = 0.013) as well. In summary, our study suggests that the risk of surgical NEC and FIP is higher in patients with blood group AB and lower in those having non-AB blood groups.

## Introduction

Necrotizing enterocolitis (NEC) and focal intestinal perforation (FIP) are two of the most common emergencies of the gastrointestinal tract in preterm infants with very low birth weight (VLBW)^1, 2^. Although FIP and NEC have been recognized as distinct entities^3, 4^, the clinical features and timing of presentation are frequently overlapping, making both diseases clinically indistinguishable in many cases^5^. NEC typically occurs in infants born between 22 and 28 weeks of gestation within the second or third week of life^6, 7^. In contrast, the gestational age of children developing FIP ranges between 22 and 27 weeks with intestinal perforation occurring within the first or second week of life^8, 9^. Moreover, infants with FIP can develop peritonitis mimicking NEC, and NEC may occur after an episode of FIP^10–13^. Both NEC and FIP culminate in necrosis of the intestinal mucosa, which leads to perforation of the bowel^14^. Neonatal mortality is lower among infants with FIP (21%) compared to those with surgical NEC (35%)^15^. For both NEC and FIP, early diagnosis and prediction of disease severity are crucial.

The etiology and pathogenesis of NEC and FIP remain poorly understood. Epidemiologic observations strongly suggest a multifactorial nature, with prematurity beings the most significant risk factor^16, 17^. The physiological immaturity of the gastrointestinal tract results in impairment of intestinal motility, digestive ability, splanchnic perfusion, and the intestinal barrier^18–21^. Risk factors for NEC include alterations of the intestinal microbiome composition^22^, formula feeding (exposure to cow milk protein)^23^, and dysregulation of the immune system^24^. In contrast, maternal chorioamnionitis^25^ and exposure to postnatal steroids with or without indomethacin treatment^26^ are associated with FIP.

In 2012, Thomson et al. showed that neonatal blood group AB is associated with an increased mortality in preterm infants with NEC^27^. Blood group antigens are not only on the surface of red blood cells but also occur in other tissues, including the intestinal surface. Moreover, they can be secreted into the lumen of the gut (and breast milk)—for ABO, for example, if the person carries another specific genetic variant in the gene *FUT2* and is a so-called “secretor”—where these sugars serve as nutrients for intestinal microbes. Blood group antigens also serve as receptors for toxins, parasites, and bacteria, facilitating their colonization or invasion. They can also serve as false receptors, preventing binding to a target tissue. ABO antibodies contribute to the innate immune system and attack some bacteria or viruses that carry ABO antigens^28^.

In this study, we utilized a large cohort study of the German Neonatal Network (GNN) consisting of 10,257 VLBW infants to investigate the potential relationships between neonatal blood group AB and risk for developing surgical FIP and NEC.

## Results

### Baseline characteristics of study population

Of the 10,257 VLBW infants enrolled in this study, 3538 (34.5%) neonates had blood group O, 4773 (46.5%) blood group A, 1332 (13.0%) blood group B, and 614 (6.0%) blood group AB. Among them, there was no difference in gestational age, birth weight, gender, mode of delivery, and antenatal corticosteroid exposure (Table 1). However, differences in blood group distribution were observed for multiple birth (blood group B [390 multiples of 1332, 29.3%] vs. blood group A [1603 multiples of 4773, 33.6%], descriptive p = 0.003). The NEC/FIP group comprised of 441 (4.3%) neonates, including 156 (35.4%) patients with blood group O, 196 (44.4%) with blood group A, 51 (11.6%) with blood group B, and 38 (8.6%) with blood group AB.

**Table 1:** Baseline characteristics of the study population, stratified by ABO blood group.

Variable	O (n = 3538)	B (n = 1332)	A (n = 4773)	non-AB (n = 9643)	AB (n = 614)	Descriptive p value AB vs. non-AB
Gestational age, wk	28.5 ± 2.7	28.5 ± 2.7	28.5 ± 2.6	28.5 ± 2.6	28.5 ± 2.7	> 0.99
Birth weight, g	1034 ± 304	1029 ± 309	1041 ± 303	1037 ± 304	1051 ± 308	0.26
Male sex	1864 (52.7)	686 (51.5)	2415 (50.6)	4965 (51.5)	301 (49.0)	0.23
Multiple birth	1238 (35.0)	390 (29.3)	1603 (33.6)	3231 (33.5)	187 (30.5)	0.12
SGA	629 (17.8)	246 (18.5)	881 (18.5)	1756 (18.2)	102 (16.6)	0.32

Values are mean with corresponding standard deviation or n (%); *SGA* small for gestational age, *NEC* necrotizing enterocolitis, *FIP* focal intestinal perforation; *For continuous variables Mann–Whitney-U test and for binary variables $\chi$^2^ test was used.

### Blood group AB and risk for NEC/FIP

**Figure 1:**
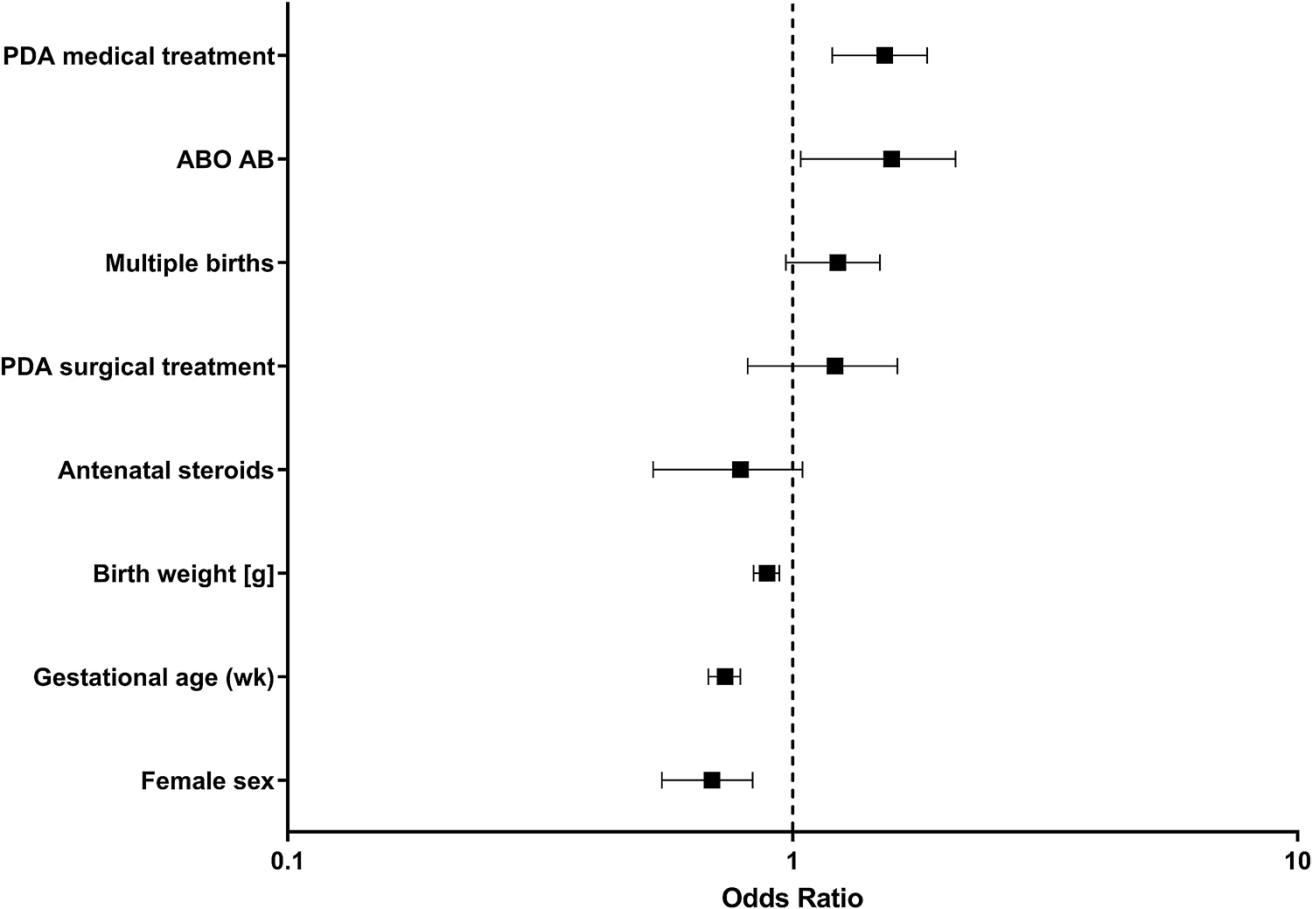
Risk for NEC/FIP—multivariable logistic regression model. Multivariable logistic regression model to adjust the effect of ABO blood group for covariates including multiple births, PDA medical treatment, surgical treatment of PDA, female sex, antenatal steroids, birth weight, and gestational age. The symbols and lines describe odds ratios and corresponding 95% confidence intervals. *NEC* necrotizing enterocolitis, *FIP* focal intestinal perforation, *PDA* patent ductus arteriosus, *RDS* respiratory distress syndrome.

In univariate analyses, preterm infants with blood group AB were at higher risk for surgical NEC/FIP compared to the non-AB-groups (OR 1.51, 95% CI 1.07–2.13, p = 0.02; absolute risk difference 2.01%, 95% CI 0.06–3.96%). When the risk for surgical NEC/FIP or death was analyzed, the difference between the AB- and non-AB-group persisted (Table 2). In a multivariable logistic regression model which included covariates such as sex, gestational age, birth weight, multiple birth, medical and surgical PDA treatments, we could observe a significantly higher risk of blood group AB for NEC/FIP when compared to non-AB-blood groups (OR 1.54, 95% CI 1.10–2.26, p = 0.01) (Fig. 1). We also found that medical treatment of PDA using indomethacin or ibuprofen was associated with increased risk for developing NEC/FIP (OR 1.50, 95% CI 1.21–1.85, p < 0.0001). In contrast, female sex (OR 0.68, 95% CI 0.55–0.83, p < 0.0001), higher gestational age (OR 0.73, 95% CI 0.68–0.78, p < 0.0001) and higher birth weight (OR 0.89, 95% CI 0.83–0.94, p < 0.0001) emerged as protective factors against NEC/FIP. As shown in Table 3, we did not detect any associations between blood group distribution and secondary outcome variables.

**Table 2:** Risk for surgical NEC/FIP in relation to blood group.

Classification	ABO blood group					AB vs. non-AB
	O n = 3538	A n = 4773	B n = 1332	Non-AB n = 9643	AB n = 614	OR (95% CI), p value; Absolute risk difference (95% CI)
Surgical NEC/FIP	156 (4.4)	196 (4.1)	51 (3.8)	403 (4.2)	38 (6.2)	1.51 (1.07–2.13), p = 0.017; 2.01% (0.06–3.96%)
Surgical NEC/FIP or death*	158 (4.5)	203 (4.3)	52 (3.9)	413 (4.3)	39 (6.4)	1.51 (1.08–2.12), p = 0.015; 2.07% (0.10–4.04%)

Values in blood group are given as n (%). *NEC* necrotizing enterocolitis, *FIP* focal intestinal perforation, Pearson´s $\chi$^2^ test, *OR* odds ratio, *CI* confidence interval. *Patients with NEC/FIP in Bell stage ≥ 2° undergoing surgical intervention (n = 441) or death without surgery related to NEC/FIP (n = 11).

**Table 3:** Secondary outcomes in relation to ABO blood group.

Variable	O n = 3538	A n = 4773	B n = 1332	Non-AB n = 9643	AB n = 614	AB vs. non-AB OR (95% CI), p value
Overall mortality	129 (3.6)	166 (3.5)	41 (3.1)	336 (3.5)	14 (2.3)	0.65 (0.38–1.11), p = 0.135
Mortality from NEC/FIP	9 (5.7%)	17 (8.4%)	2 (3.8)	28 (6.8)	3 (7.7)	1.68 (0.51–5.56), p = 0.385
IVH	620 (17.6)	857 (18.0)	244 (18.3)	1721 (17.8)	111 (18.1)	1.03 (0.83–1.27), p = 0.798
PVL	89 (2.5)	145 (3.0)	42 (3.2)	276 (2.9)	12 (2.0)	0.67 (0.37–1.21), p = 0.186
PDA surgical treatment	132 (3.7)	190 (4.0)	45 (3.4)	367 (3.8)	21 (3.4)	0.89 (0.57–1.40), p = 0.627
PDA medical treatment	1001 (28.3)	1324 (27.8)	344 (25.9)	2669 (27.7)	160 (26.1)	0.92 (0.76–1.10), p = 0.384
BPD	643 (18.2)	791 (16.6)	234 (17.6)	1668 (17.3)	102 (16.6)	0.95 (0.76–1.18), p = 0.663
ROP	118 (3.4)	180 (3.8)	39 (3.0)	337 (3.5)	22 (3.6)	1.02 (0.66–1.59), p = 0.908
Sepsis	436 (12.3)	573 (12.0)	150 (11.3)	1159 (12.0)	83 (13.5)	1.14 (0.90–1.45), p = 0.269

Values are n (%); *NEC* necrotizing enterocolitis, *FIP* focal intestinal perforation, *IVH* intraventricular hemorrhage, *PVL* periventricular leukomalacia**,** *PDA* patent ductus arteriosus**,** *BPD* bronchopulmonary dysplasia, *ROP* retinopathy of prematurity.

## Discussion

We found that neonates with blood group AB are at increased risk of developing surgical NEC and FIP compared to individuals with non-AB-blood groups. Consequently, blood group AB may be considered as a novel risk factor for developing NEC/FIP in VLBW infants besides the well-known factors including gestational age, hemodynamically relevant PDA, and male gender. The awareness of this association should contribute to earlier diagnosis and treatment of both potentially devastating diseases. However, we are aware that blood group AB is infrequent, and only a small proportion of VLBW neonates can be potentially observed more closely.

The ABO blood group system comprises the carbohydrate molecules (A, B, and H antigens) that are expressed on the extracellular surface of red blood cells as well as a variety of human tissues, including platelets, leukocytes, plasma proteins, the vascular endothelium, and the mucus layer^29^. These ABO antigens locating on tissues are histo-blood group antigens (HBGAs). The proposed functions of HBGAs are mostly cell-cell recognition, facilitation of intracellular uptake, signal transduction, enzymatic activity, and glycocalyx formation^30^. Furthermore, blood group antigens serve as receptors and coreceptors for bacteria, parasites, and viruses, contributing to host susceptibility or resistance to various infectious and non-infectious diseases. Finally, several blood groups can additionally modify the innate immune response to infection^28^.

To date, only one study investigated the effect of neonatal and maternal blood group distribution on NEC mortality. In 2012, Thomson and colleagues analyzed 276 neonates with Bell stage II-III NEC and found that neonates with blood group AB (n = 60, 21.7%) had a significantly higher risk of mortality from NEC compared to other blood groups (Hazard ratio 2.87, 95% CI 1.40–5.89, p = 0.003)^27^. In contrast, in our cohort, we could not confirm an association between blood group and risk of mortality related to NEC/FIP. These discordant findings can be partially explained by the fact that the mortality from NEC/FIP in our patient cohort was very low (n = 31, 7.0% of all infants with surgery for FIP or NEC) compared to those reported by others (up to 32%^31, 32^), which may have affected the statistical power and validity.

In the intestine, blood group antigens are found on red blood cells, mucosal epithelia, in the mucus layer, and in biologic fluids (saliva, intestinal contents, milk)^33–35^. Individuals who are non-secretors of blood group antigens do not have ABH antigens expressed on the mucosal secretions and surfaces^36^. HBGAs are recognized as receptors by numerous pathogens, including, noroviruses^37–39^, rotavirus^40–42^, coronaviruses^43^, and *Helicobacter pylori*^44^. H. pylori strains, for example, binds to all three ABH blood group antigens via the Blood group Antigen Binding Adhesin^45^. The most well-known example of blood-group-associated diseases is cholera, which affects predominantly individuals with blood group O leading to more severe symptoms than those with other blood groups^46^. Although the primary receptor of the cholera toxin is the GM1 ganglioside, it can also bind to HBGAs, facilitating adhesion on the surface of the enterocytes^47^. More recently, a study conducted by the Severe Covid-19 GWAS Group has shown that individuals with blood group A are at higher risk for developing symptomatic Covid-19 with respiratory failure compared to other blood groups suggesting the potential involvement of the ABO blood group system in the pathogenesis of Covid-19 infection^48^. Furthermore, there is increasing evidence for a HBGA-mediated modulation of the intestinal microbiome. For example, certain lactobacilli such as L. gasseri OLL2755 or L. gasseri OLL2877 bind to HBGAs^49^. Healthy subjects with different HBGA were shown to possess distinct profiles of fecal microbiota with a blood group secretor status being associated with less diversity at higher-order taxa and an expansion of distinct bacterial families, e.g., *Clostridiales*, in the presence of blood group A antigens^50, 51^.

While less is known about an association between FIP and the pattern of gut colonization, multiple evidence has been provided that NEC is preceded by dysbiotic microbiota profiles^52, 53^. The impact of HBGAs on the shaping of the gut microbiota might represent the pathogenetic clue explaining the increased occurrence of NEC in neonates with blood group AB and should be further elucidated in future studies.

Finally, whether packed red blood cell transfusion increases the risk for NEC (so-called transfusion-associated necrotizing enterocolitis) is subject of ongoing discussions^54, 55^. As blood products with blood group AB are scarce and individuals with blood group AB are universal recipients, blood group compatible but not identical transfusions might be more frequent in patients with blood group AB compared to individuals with non-AB. Furthermore, in some institutions, preterm infants are transfused with blood group O, irrespective of their own blood group^27^. As individuals with blood group AB express A and B antigens in various tissues, the transfusion of blood products containing isoagglutinins (i.e., anti-A or anti-B antibodies) might augment the risk for surgical NEC/FIP in preterm infants carrying AB antigens. However, to test this hypothesis, register studies in VLBW preterm infants should include information on the type (blood group identical or blood-group compatible) and timing (transfusion bevor NEC or after) of red blood cell transfusions.

We are aware of several limitations of our study, including the low prevalence of blood group AB among neonates with NEC/FIP (n = 39, 5.9%). Furthermore, unrecognized confounders such as the prenatal course, center-specific protocols for feeding advancement, and availability of donor milk in some centers might have influenced the results of our study. Overall the association of blood group AB and NEC/FIP needs to be tested in other populations, e.g., in cohorts with a higher prevalence of blood group AB. We are also aware that the intra-operative findings and histopathology of FIP are different from that of NEC. However, histopathological data is lacking in the GNN cohort, thus making precise differentiation between both diseases impossible. Moreover, as both entities have an overlapping clinical presentation and there is currently no well-defined set of objective criteria to intra-operatively distinguish NEC from FIP, it is possible that in the central GNN database, a code for “NEC” was chosen instead of “FIP” and vice versa. To overcome both limitations, we used “surgical FIP/NEC” as a robust outcome parameter.

On the other hand, this study has several strengths. Our cohort is quite homogeneous in ethnicity, the degree of prematurity, and the clinical treatment strategies. To the best of our knowledge, we present the first study investigating the association between ABO blood group and risk for NEC/FIP in a large, well-defined cohort of VLBW infants with stringent criteria for NEC/FIP (both requiring surgical intervention). Furthermore, we also excluded neonates with the diagnosis “medical NEC” (not requiring surgery), as this condition is difficult to distinguish from other disease entities. Another strength of our study is that the blood groups were determined genetically, which represents the most accurate method.

## Conclusion

Here, we demonstrate that among VLBW infants, blood group AB is associated with an increased risk of NEC/FIP. Therefore, blood group AB may be considered as an additional risk factor for NEC/FIP. The underlying mechanism of the blood group AB in NEC/FIP development, including potential interactions between intestinal bacteria and HBGAs needs to be further elucidated.

## Methods

### Study cohort

Subjects were enrolled in the prospective cohort study of the German Neonatal Network (GNN) between 2009 and 2016 by 43 participating tertiary German neonatal intensive care units. Infants with a birth weight below 1500 g and gestational age below 37 weeks who were admitted to one of the participating centers were eligible for participation. Written informed consent was obtained from parents for research and publication of data. The ethics committee at the University of Lübeck and at all participating hospital approved the study: Medical University of Hannover; Regional Medical board of Westfalen-Lippe and the University of Münster; Heinrich Heine University Düsseldorf; University of Rostock; University of Duisburg-Essen; Ruhr University Bochum; University of Greifswald; University of Cologne; University of Regensburg; Otto von Guericke University Magdeburg; Witten/Herdecke University; Dresden University of Technology; University of Kiel; Heidelberg University (University Hospital Mannheim); University of Tübingen; RWTH Aachen University; Martin Luther University of Halle-Wittenberg; University of Göttingen; University Medical Center Freiburg; University of Bonn; University of Jena; University of Erlangen-Nuremberg; University of Ulm; Goethe University Frankfurt; Technical University of Munich; University of Würzburg; and the Regional Medical boards of Hessen, Hamburg, Bayern, Berlin, Baden-Württemberg, Saarland, Nordrhein, Schleswig–Holstein, Bremen, and Niedersachsen. All experimental protocols were performed in accordance with relevant guidelines and approved by the ethics committee of the University of Lübeck. Neonatal data were collected locally by attending physicians and sent to the coordinating center in Lübeck. Data quality was ensured by regular onsite monitoring of participating centers. All data were entered in a database by health record administrators at the main GNN office at the University of Lübeck, Germany. Early neonatal death, parental refusal, or parents not asked for permission of their infant to participate were reasons for non-enrollment. Data were collected from every infant on important maternal, fetal, and neonatal parameters, including maternal ethnic origin, sex, singleton vs. multiple births, fetal malformation, pharmacological or surgical treated patent ductus arteriosus (PDA), sepsis, bronchopulmonary dysplasia (BPD), intraventricular hemorrhage (IVH), periventricular leukomalacia (PVL).

### Definitions of outcome parameters

**Primary outcome** was the frequency of NEC/FIP in infants with blood group AB (n = 614, 6.0%) compared to 9643 (94.0%) non-AB neonates.

**Secondary outcome parameters** included NEC/FIP related and overall mortality, occurrence of complications such as IVH, PVL, BPD, retinopathy of prematurity (ROP) or sepsis, and treatment modalities such as surgical or medical treatment of PDA according to blood group distribution in infants with blood group AB versus non-AB.

**Sepsis** was either clinical sepsis or blood culture-proven sepsis. According to our previous publications^56, 57^, clinical sepsis was defined as sepsis with at least two signs (temperature > 38.0 °C or < 36.5 °C, tachycardia > 200/min, new-onset or increased frequency of bradycardia or apnea, hyperglycemia > 7.8 mmol/l, base excess <  − 10 mval/l, change in skin color, increased oxygen requirements) and one laboratory sign (C-reactive protein > 10 mg/l, immature/neutrophil ratio > 0.2, white blood cell count < 5/nl, platelet count < 100/nl) and antibiotic treatment for ≥ 5 days, but no detection of a causative agent in the blood culture. Blood-culture confirmed sepsis was defined as clinical sepsis with proof of a causative agent in the blood culture^58^.

**Intraventricular hemorrhage** (IVH): graded according to the Papile classification^59^.

**Periventricular leukomalacia** (PVL): cystic degeneration of white matter near the lateral ventricles (ultrasound or MR).

**Retinopathy of prematurity** (ROP): ROP requiring any intervention (e.g., cryotherapy, laser therapy, or anti-VEGF treatment).

**Focal intestinal perforation** (FIP): focal intestinal perforation with the need for laparotomy.

**Surgical Necrotizing enterocolitis** (NEC): clinical NEC with the need for laparotomy with or without resection of the necrotic gut and the macroscopic diagnosis of NEC. The attending pediatric surgeon differentiated in NEC or FIP.

**FIP/NEC-related mortality** was the subgroup of children with diagnosed NEC in Bell stages II or III without surgery but death (n = 11). In monitoring visits, the probable cause of death was determined.

**Small for gestational age** (SGA): Birth weight below the 10^th^ percentile according to Voigt et al.^60^.

**Patent ductus arteriosus (PDA) surgery**: surgical ligation of PDA.

**Patent ductus arteriosus (PDA) pharmacological treatment:** therapy with indomethacin or ibuprofen and PDA.

**Bronchopulmonary dysplasia** (BPD): need for ventilation support or oxygen supplementation at 36 weeks corrected age.

**Death** was defined as mortality during the primary stay in the hospital.

### Genotyping

Patients’ data and samples were coded before genotyping and analysis. Umbilical cord tissue of participating infants was collected after birth and stored at − 20 °C until transfer to the University of Lübeck. Genotyping was performed using the previously described methodology^56^. DNA was extracted using standard protocols for commercial DNA purification kits (Gentra Puregene Tissue Kit, Qiagen, Hilden, Germany). Chip genotyping was done by the Cologne Center for Genomics using ‘Axiom CEU’ (Affymetrix, Santa Clara, California, USA) or the Institute of Clinical Molecular Biology Kiel using ‘Global Screening Array (GSA)’ (Illumina, San Diego, California, USA). After chip genotyping of approximately 500,000 single nucleotide polymorphisms (SNPs) per infant and quality control of samples and SNPs, additional SNPs were imputed. The ABO gene was determined according to two SNPs (rs8176746 and rs8176719) as described by Groot et al.^61^. The SNP rs8176719 was not available for samples genotyped on the Illumina chip and the proxy SNP rs8176645 was used instead.

### Statistical analysis

Study population was grouped by the blood groups, A, B, O, or AB and additionally non-AB (A, B, and O). Groups were tested with $\chi$^2^ test and Mann–Whitney-U-test to describe differences descriptively. The association of non-AB vs. AB with the predefined outcomes (surgical treatment of NEC/FIP, and surgical treatment of NEC/FIP or death) was determined by OR, absolute risk difference, corresponding confidence interval, and p value from $\chi$^2^ test. In addition, the association of the blood groups non-AB vs. AB on several secondary outcomes (overall mortality, mortality from NEC/FIP, IVH, PVL, surgical treatment of PDA, medical treatment of PDA, BPD, ROP, sepsis) was determined by OR, absolute risk difference and corresponding confidence interval. The association of non-AB vs. AB with surgical treatment of NEC/FIP was calculated in multivariable logistic regression analyses with adjustment for known influencing variables (gestational age, birth weight as continuous variables), and further covariates (antenatal steroids, sex, drug treatment of PDA, surgical treatment of PDA, and multiple births). The type I error level was set to 0.05. Data analysis was performed using SPSS 25.0 (IBM, Munich, Germany). Data visualization was done by GraphPad Prism version 6.21 for Windows (GraphPad Software, San Diego, CA).

## Data Availability

The datasets generated and analyzed during the current study are not publicly available but can be reviewed on reasonable request by Wolfgang Göpel (Head of Studies, GNN).
